# Enhanced mGluR_5_ intracellular activity causes psychiatric alterations in Niemann Pick type C disease

**DOI:** 10.1038/s41419-024-07158-8

**Published:** 2024-10-23

**Authors:** Ana Toledano-Zaragoza, Violeta Enriquez-Zarralanga, Sara Naya-Forcano, Víctor Briz, Rocío Alfaro-Ruíz, Miguel Parra-Martínez, Daniel N. Mitroi, Rafael Luján, José A. Esteban, María Dolores Ledesma

**Affiliations:** 1 Centro Biología Molecular Severo Ochoa (CSIC-UAM), Madrid, Spain; 2 Centro Nacional de Sanidad Ambiental, Instituto Salud Carlos III, Majadahonda, Spain; 3 Synaptic Structure Laboratory, Instituto de Investigación en Discapacidades Neurológicas (IDINE), Facultad de Medicina, Universidad de Castilla-La Mancha, Albacete, Spain

**Keywords:** Lipid-storage diseases, Pathogenesis

## Abstract

Niemann-Pick disease Type C (NPC) is caused by mutations in the cholesterol transport protein NPC1 leading to the endolysosomal accumulation of the lipid and to psychiatric alterations. Using an NPC mouse model (Npc1^nmf164^) we show aberrant mGluR_5_ lysosomal accumulation and reduction at plasma membrane in NPC1 deficient neurons. This phenotype was induced in wild-type (wt) neurons by genetic and pharmacological NPC1 silencing. Extraction of cholesterol normalized mGluR_5_ distribution in NPC1-deficient neurons. Intracellular accumulation of mGluR_5_ was functionally active leading to enhanced mGluR-dependent long-term depression (mGluR-LTD) in Npc1^nmf164^ hippocampal slices. mGluR-LTD was lower or higher in Npc1^nmf164^ slices compared with wt when stimulated with non-membrane-permeable or membrane-permeable mGluR_5_ agonists, respectively. Oral treatment with the mGluR_5_ antagonist 2-chloro-4-((2,5-dimethyl-1-(4-(trifluoromethoxy)phenyl)-1*H*-imidazol-4-yl)ethynyl)pyridine (CTEP) reduced mGluR-LTD and ameliorated psychiatric anomalies in the Npc1^nmf164^ mice. Increased neuronal mGluR_5_ levels were found in an NPC patient. These results implicate mGluR_5_ alterations in NPC psychiatric condition and provide a new therapeutic strategy that might help patients suffering from this devastating disease.

## Introduction

Niemann Pick disease type C (NPC) is a lysosomal storage disorder caused by mutations in the cholesterol transport protein NPC1 [1]. Despite having visceral involvement NPC is primarily a neurological disease that is classified as infantile, juvenile or adult based on the timing of neurological symptom onset [2]. Besides cognitive impairment, psychiatric alterations are frequent in NPC patients and may lead to psychosis and dementia [3]; these symptoms point to synaptic anomalies as key events in NPC. Presynaptic defects [4] and changes in neurotransmitter levels [5] have been described in the NPC1-null mouse, a model which mimics the most aggressive early-onset forms of NPC [6]. Reduced levels of the voltage-gated potassium channels leading to hyperexcitability have been observed in Npc1-deficient cultured neurons [7]. Studies performed in the delayed onset Npc1^nmf164^ mouse model, which carries an Npc1 mutation in a region commonly mutated in human disease [8], showed a critical role for NPC1 in synaptic plasticity [9]. This protein enables mobilization of the cholesterol-modifying enzyme cholesterol-24S-hydroxylase (Cyp46) to the neuronal surface, allowing cholesterol release from synaptic membranes and the surface delivery of glutamate receptors of the α-amino-3-hydroxy-5-methyl-4-isoxazole propionic acid (AMPA) type. This process is necessary for long-term potentiation (LTP) [9], which underlies learning and memory. NPC1 deficiency caused the accumulation of cholesterol in synapsis and impaired Cyp46 and AMPA receptor surface delivery, LTP progression and memory in Npc1^nmf164^ mice [9].

Synaptic plasticity is not only important for learning and memory but also controls emotions and behavior. Particularly relevant in this context are the alterations in the metabotropic glutamate receptor type 5 (mGluR_5_) [10], which have been associated with a number of psychiatric conditions including depression, bipolar disorder, anxiety, schizophrenia and autism [11]. Drugs that have the ability to modulate mGluR_5_ function have anxiolytic and anti-depressive effects with a relatively benign safety profile [11]. mGluR_5_ is a G-protein-coupled receptor involved in the fine-tuning of glutamatergic synaptic transmission and it mediates a specific synaptic plasticity event known as mGluR-dependent long term depression (mGluR-LTD) [10, 11]. mGluR_5_ shows cell surface and intracellular distribution in the neurons, and the signaling triggered by receptor activation depends on its subcellular localization [12]. Thus, trafficking of mGluR_5_ is crucial for its proper function and is tightly controlled. A significant pool of mGluR_5_ localizes to lipid raft membrane domains, which are enriched in cholesterol and mediate the internalization of the receptor [13]. These results suggest that the amount of cholesterol is important to control the level and distribution, and therefore function, of mGluR_5_.

In this work we have confirmed the hypothesis that altered cholesterol distribution resulting from NPC1 deficiency affects mGluR_5_ levels, localization and function, leading to psychiatric anomalies in NPC, which could be prevented by mGluR_5_ pharmacological modulation.

## Results

### NPC1 deficiency alters the levels and subcellular localization of mGluR_5_ in neurons

**Fig. 1:**
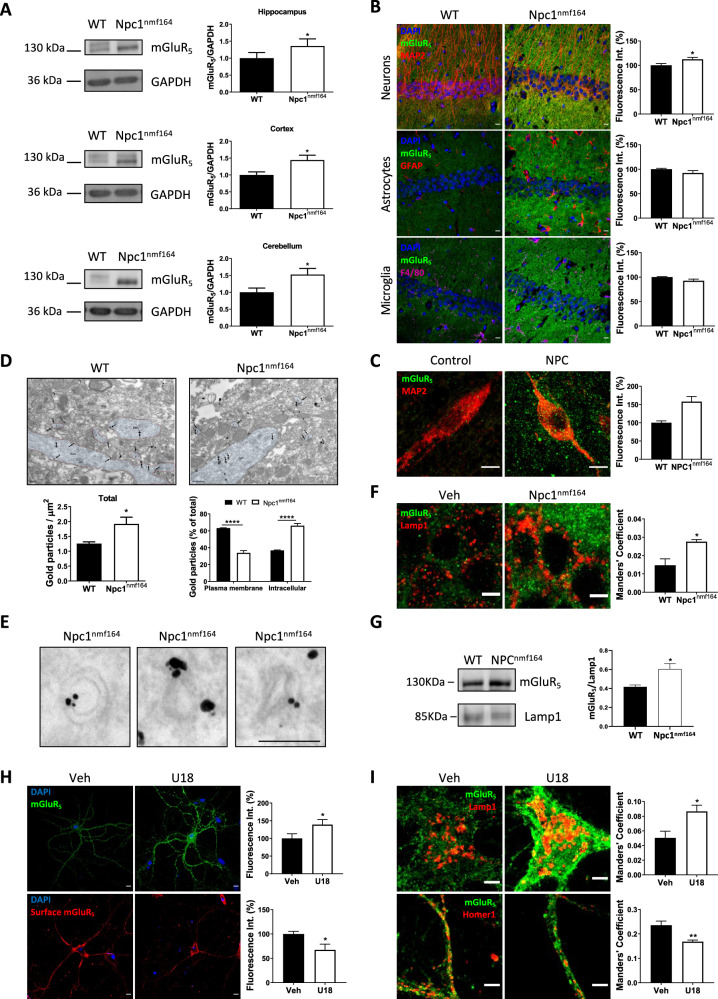
Increased levels and altered localization of mGluR_5_ in NPC1 deficient neurons. **A** Western blot analysis of mGluR_5_ and GAPDH levels in extracts from the hippocampus, cortex and cerebellum of age-matched wt and Npc1^nmf164^ mice. Graphs show mean ± SEM levels of mGluR_5_ normalized to GAPDH levels that were used as loading control (*P*_*hippoccampus*_ = 0.0482; *P*_*cortex*_ = 0.0272; *P*_*cerebellum*_ = 0.0359) *n* = 6; Student’s *t* test). **B** Immunofluorescence analysis (IFA) of mGluR_5_ co-stained with markers for neurons (MAP2), astrocytes (GFAP) or microglia (F4/80) in the hippocampus of wt and Npc1^nmf164^ mice. Graphs show mean ± SEM fluorescence intensity of mGluR_5_ in each type of cell as a percentage of the levels in the wt images. (*P*_*neurons*_ = 0.0445; *n* = 5; Student’s *t* test). Scale bar 10 µm. **C** IFA of mGluR_5_ and MAP2 (marker for soma and dendrites of neurons) in the hippocampus of control child and NPC patient. Graph shows mean ± SEM fluorescence intensity as a percentage of the levels in the control child (*n* = 20 neurons). Scale bar 10 µm. **D** Immunoelectron microscopy showing mGluR_5_ in pyramidal neurons of the hippocampus from wt and Npc1^nmf164^ mice. Left graph: mean ± SEM number of mGluR_5_-associated gold particles per μm^2^ *P* = 0.0304; *n* = 4; Student’s *t* test); Right graph: mean ± SEM number of plasma membrane or intracellular mGluR_5_-associated gold particles as a percentage of the total gold particles (*P*_*plasma membrane*_ < 0.0001; *P*_*intracellular*_ < 0.0001; *n* = 4; 2-way ANOVA Bonferroni *post-hoc*). **E** Crops of immunoelectron micrographs showing mGluR_5_- associated gold particles in membranous structures compatible with endolysosomes in pyramidal neurons from Npc1^nmf164^ mice. Scale bar 250 nm. **F** IFA of mGluR_5_ co-stained with the endolysosomal marker Lamp1 in a pyramidal neuron of the hippocampus from wt and Npc1^nmf164^ mice. Graph shows mean ± SEM Manders’ coefficient of co-localization between mGluR_5_ and Lamp1 (*P* = 0.0134; *n* = 4; Student’s *t* test). Scale bar 5 µm. **G** Western blot analysis of mGluR_5_ and Lamp1 levels in lysosomal-enriched fractions from the brain of wt and Npc1^nmf164^ mice. Graphs show mean ± SEM levels of mGluR5 normalized to Lamp1 (*P* = 0.0381; *n* = 3; Paired *t* test). **H**. IFA of mGluR_5_ in cultured hippocampal neurons permeabilized (upper panels, Total mGluR_5_) or not (lower panels, Surface mGluR_5_) from wt mice treated with vehicle (Veh) or with U18 (NPC1 inhibitor). Graphs show mean ± SEM fluorescence intensity as percentage of the levels in the wt images (*P*_*mGluR5*_ = 0.0197; *P*_*surface mGluR5*_ = 0.0363 *n* = 5; paired Student’s *t* test). Scale bar 10 µm. **I**. IFA of mGluR_5_ co-stained with the markers of endolysosomes Lamp1 or of surface Homer1 in cultured hippocampal neurons from wt mice treated with U18 or Veh. Graphs show mean ± SEM Manders’ coefficient of co-localization between mGluR_5_ and Lamp1 or Homer1 (*P*_*mGluR5-Lamp1*_ = 0.0182; *P*_*mGluR5-Homer1*_ = 0.0071 *n* = 5–6; paired Student’s *t* test). Scale bar 5 µm.

Protein expression of mGluR_5_ was determined by Western blot in the hippocampus, cortex and cerebellum of wild-type (wt) and Npc1^nmf164^ mice at 2.5 months of age. At this age, intracellular accumulation of cholesterol and symptoms of the disease already manifest in the Npc1^nmf164^ mice. There was an increase in mGluR_5_ protein in all areas analyzed: hippocampus (36%), cortex (44%) and cerebellum (52%), of the Npc1^nmf164^ compared with the wt controls (Fig. 1A, uncropped blots in Supplementary Fig. S10). Quantification of mGluR_5_ mRNA by qRT-PCR did not show differences in gene expression in the hippocampus or cortex, but indicated a reduction in the cerebellum (Supplementary Fig. S1), which could be explained by the cerebellar cell death that occurs at early disease stages [14]. Given that mGluR_5_ expression is particularly abundant in the hippocampus [15, 16] and the alterations found in the NPC mouse model, we focused on this brain area for subsequent analysis. To determine whether the observed changes differentially affected neurons and glial cells, we next measured the levels of mGluR_5_ in each cell type by double immunofluorescence analysis (IFA) using markers for neurons (MAP2, which labels the neuronal soma and dendrites), astrocytes (GFAP) or microglia (F4/80). Levels of mGluR_5_ were significantly increased in neurons (12%) but unchanged in microglia or astrocytes from Npc1^nmf164^ mice compared with wt animals (Fig. 1B). To determine whether these observed neuronal mGluR_5_ alterations are consistent with the clinical disease, we compared fixed brain tissue from a patient with NPC and a control child. mGluR_5_ IFA showed a 57% increase in the receptor levels in pyramidal hippocampal neurons of the NPC patient relative to the control (Fig. 1C). While these observations are not conclusive, since they were made in a single case due to the difficulty in obtaining fixed brain samples from patients of this rare disease, they suggest common mGluR_5_ alterations in NPC-affected humans and mice. To gain insight into the subcellular distribution of mGluR_5_ we performed immunoelectron microscopy in pyramidal neurons collected from the hippocampus of 2.5-month-old Npc1^nmf164^ and wt mice. Consistent with our previous results with Western blot and IFA, this analysis showed an increase (52%) in total mGluR_5_ in the Npc1^nmf164^ mice compared with wt controls, and also indicated changes in receptor distribution. Whereas the wt mice had 63,2% of mGluR_5_-associated gold particles at the plasma membrane and 36,8% intracellular, the Npc1^nmf164^ mice had an inverted ratio (34,2% at plasma membrane and 65,8% intracellular) (Fig. 1D). mGluR_5_-associated gold particles were enriched in membranous structures compatible with endolysosomes (Fig. 1E). Double IFA using antibodies against mGluR_5_ and the endolysosomal marker Lamp1 showed the increased presence of the receptor in endolysosomes in hippocampal neurons from the Npc1^nmf164^ mice compared with wt controls (Fig. 1F). The elevated levels of mGluR_5_ in lysosomes were also confirmed by biochemical isolation of these organelles from the mouse brain (Supplementary Fig. S2). Western blot showed 1.5-fold higher amount of mGluR_5_ in the lysosomal-enriched fraction from the brain of Npc1^nmf164^ mice compared to wt mice (Fig. 1G, uncropped blots in Supplementary Fig. S10).

**Fig. 2:**
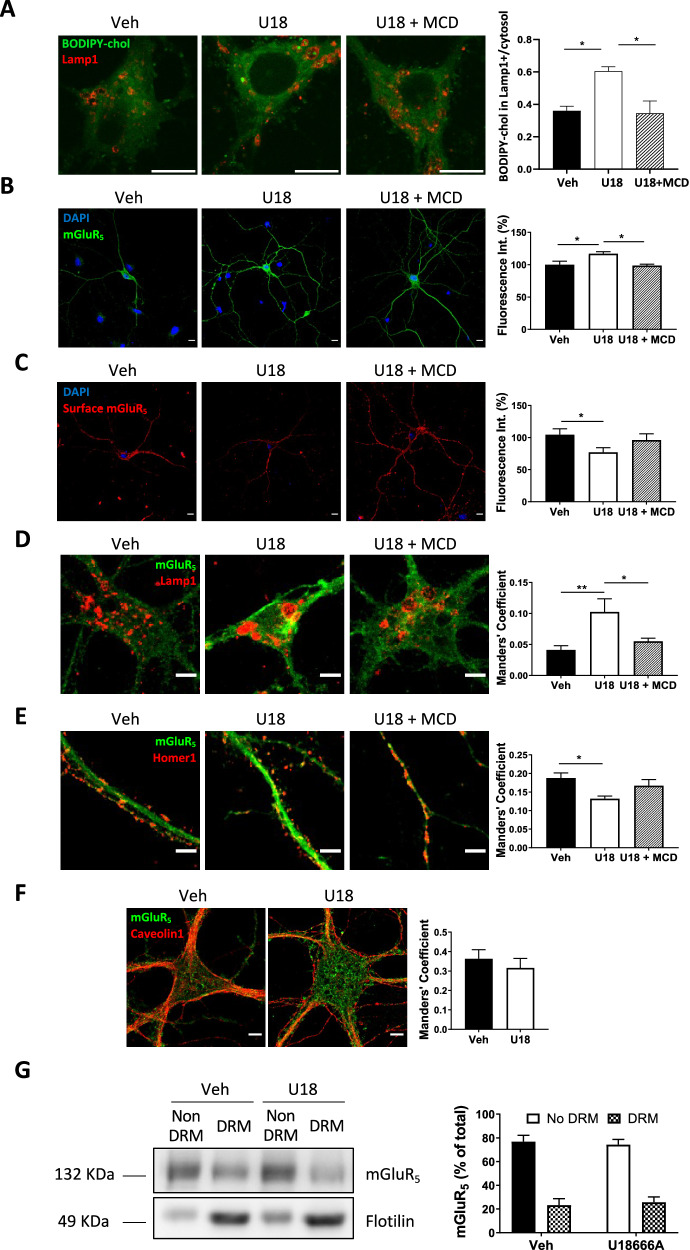
Cholesterol alterations induce mGluR_5_ changes upon NPC1 deficiency. **A** Image of BODIPY-cholesterol and Lamp1 staining in cultured neurons from wt mice treated with Veh, U18 alone or with U18 plus MCD. Graph shows mean ± SEM BODIPY-cholesterol fluorescence intensity (a.u.) in Lamp1 positive area compared to cytosol (*P*_*Veh-U18*_ = 0.0383; *P*_*U18-U18+MCD*_ = 0.0290; *n* = 3; Paired two-tailed *t* test). Scale bar 5 µm. **B**–**E** IFA of mGluR_5_ in cultured neurons from wt mice treated with Veh, U18 alone or with U18 plus MCD. **B** mGluR_5_ total staining in permeabilized neurons. **C** mGluR_5_ surface staining using anti-mGluR_5_ N-terminal in non -permeabilized neurons. **D** mGluR_5_ co-staining with Lamp1. **E** mGluR_5_ co-staining with Homer 1. Graphs in (**B**, **C**) show mean ± SEM mGluR_5_ fluorescence intensity as a percentage of the levels in the neurons treated with Veh that were considered 100% (*P*_*B. Veh-U18*_ = 0.0243; *P*_*B. U18-U18+MCD*_ = 0.0378; *P*_*C. Veh-U18*_ = 0.0471; *n* = 4–6; Grouped one-way ANOVA Bonferroni *post hoc*). Scale bar 10 µm. Graphs in (**D**, **E**) show mean ± SEM Manders’ coefficient of co-localization between mGluR_5_ and Lamp1 or Homer1 (*P*_*D. Veh-U18*_ = 0.0094; *P*_*D. U18-U18+MCD*_ = 0.0314; *P*_*E. Veh-U18*_ = 0.0419; *n* = 4–6; Grouped one-way ANOVA Bonferroni *post hoc*). Scale bar 5 µm. **F** IFA of mGluR_5_ co-stained with the raft marker caveolin1 in cultured neurons from wt mice treated with Veh or with U18. Graphs show mean ± SEM Manders’ coefficient of co-localization between mGluR_5_ and caveolin1 (*n* = 4). Scale 10 µm. **G** Western blot showing mGluR_5_ in the supernatant (detergent-resistant membrane; DRM) or pellet (non DRM) obtained after cold detergent extraction in cultured neurons from wt mice treated with U18 or Veh. The DRM canonical marker flotilin was used as control for the isolation protocol. Graph shows mean ± SEM levels of mGluR_5_ expressed as a percentage of the total levels (*n* = 6; Two-way ANOVA Bonferroni post hoc).

To assess the direct relationship between mGluR_5_ alterations and NPC1, we induced NPC1 deficiency in primary cultured neurons from wt mice by both genetic and pharmacological means. Gene silencing was carried out by lentiviral expression of an Npc1-specific shRNA, resulting in increased total mGluR_5_ and an altered distribution, diminishing its presence at the cell surface while increasing it in Lamp1-positive endolysosomal compartments (Supplementary Fig. S3). Similar results were obtained in primary cultured neurons from Npc1^nmf164^ mice (Supplementary Fig. S4). Pharmacological inhibition of NPC1 with U18666A (U18), which mimics the NPC phenotype by accumulating cholesterol in the endolysosomal compartment [17] (see also Fig. 2A), also resulted in increased total mGluR_5_ levels (38.9%) (Fig. 1H) and redistribution. Surface mGluR_5_ expression was reduced by 32.5% in U18-treated wt neurons compared with untreated controls (Fig. 1H), and co-localization of mGluR_5_ with its plasma membrane interactor Homer1 was diminished by 1.4-fold (Fig. 1I). In contrast, we observed a 1.6-fold increase in co-localization of mGluR_5_ with the endolysosomal marker Lamp1 (Fig. 1I). To better characterize the changes in the intracellular distribution of mGluR_5_ upon NPC1 deficiency we performed co-localization analyses in U18-treated wt neurons using markers for the nucleus (nuclear pore), endoplasmic reticulum (calnexin), Golgi apparatus (GM130), mitochondria (complex IV) and early endosomes (EEA1). NPC1 inhibition did not alter mGluR_5_ levels in any of the tested intracellular compartments (Supplementary Fig. S5). Furthermore, U18 treatment of cultured wt astrocytes did not alter mGluR_5_ expression (Supplementary Fig. S6), consistent with our observations in glial cells from the brains of Npc1^nmf164^ mice (see Fig. 1B).

### Cholesterol alterations induce mGluR_5_ changes upon NPC1 deficiency

To determine whether changes in cholesterol homeostasis were responsible for the alteration in neuronal mGluR_5_ levels and distribution, we modulated this lipid in cultured primary neurons. Compared to vehicle-treated wt neurons, U18-incubated wt neurons showed 1.70-fold higher cholesterol levels in Lamp1 positive organelles, as evidenced by the fluorescent cholesterol tracer boron-dipyrromethene (BODIPY)-cholesterol (Fig. 2A). Treatment of U18-incubated wt neurons with the cholesterol extracting drug methyl-β-cyclodextrin (MCD) normalized cholesterol levels in Lamp1 positive organelles (0.94-fold compared to vehicle-treated wt neurons) (Fig. 2A). MCD treatment also reduced total levels of mGluR_5_ by 15.7% (Fig. 2B) and increased surface receptor by 24.7% compared with U18-treated wt neurons not receiving MCD (Fig. 2C). MCD treatment normalized the co-localization of mGluR_5_ with Lamp1 (Fig. 2D) and Homer 1 (Fig. 2E) in U18-treated wt neurons. Since cholesterol accumulates in NPC1-deficient lysosomes and compromises their function [18], we hypothesized that lysosomal blockage may lead to mGluR_5_ alterations similar to those seen with NPC1 inhibition. To test this, we cultured wt neurons with the lysosomal inhibitor bafilomycin A1 (Baf-A1), which increased total mGluR_5_ by 22.8% and enhanced its presence in Lamp1-positive endolysosomes by 1.75-fold compared with untreated controls (Supplementary Fig. S7). Since it has been reported that cholesterol-enriched raft membrane domains regulate mGluR_5_ trafficking [13], we investigated mGluR_5_ raft partitioning by microscopy and biochemical means. Co-localization of mGluR_5_ with the raft marker caveolin1 was not altered by NPC1 inhibition in cultured wt neurons, compared with vehicle (Veh)-treated controls (Fig. 2F). Moreover, the amount of mGluR_5_ in detergent-resistant membranes (DRMs) corresponding to rafts was also similar regardless of NPC1 inhibition (Fig. 2G).

### NPC1 deficiency leads to aberrantly high mGluR_5_ function

**Fig. 3:**
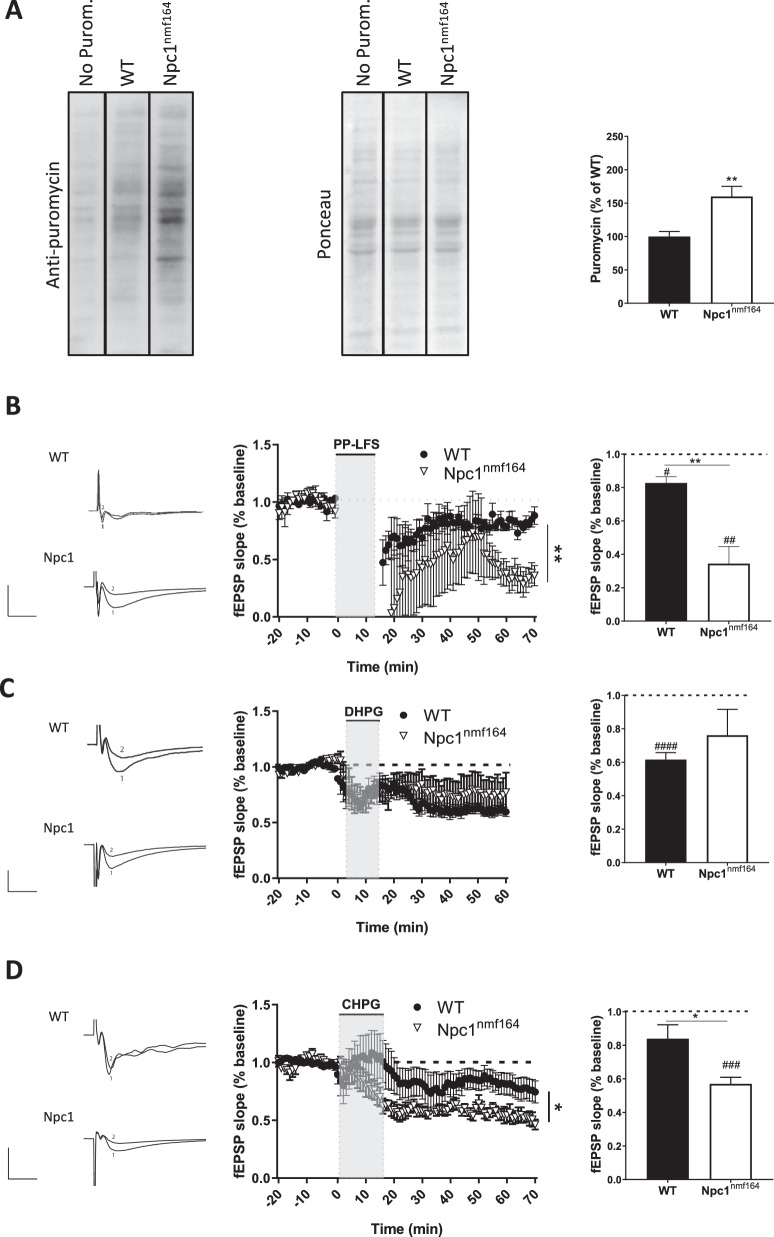
Altered mGluR_5_ function upon NPC1 deficiency. **A** Western blot showing puromycin in the SUnSET assay in hippocampal slices from wt and Npc1^nmf164^ mice. Graph shows mean ± SEM total intensity associated with puromycin as a percentage of the wt values (*P* = 0.0011; *n* = 17–18; Student’s *t* test). **B** mGluR-LTD expressed as excitatory postsynaptic field responses (fEPSP) registered in the CA1 pyramidal region of wt and Npc1^nmf164^ mice. Graphs represent fEPSP slope over baseline (left) or mGluR-LTD expressed as mean ± SEM percentage of fEPSP slope over baseline from minute 45 to minute 55 of the LTD recording (right) ((*P*_*WT Veh*_ = 0.0110; *P*_*Npc1 Veh*_ = 0.0080; *P*_*WT-Npc1*_ = 0.0004; *n* = 5 slices from 5 WT mice and *n* = 5 slices from 5 Npc1^nmf164^ mice; Student’s *t* test*;* # comparison of baseline *versus* LTD for the same genotype; *comparison between different genotypes). Representative traces of responses to stimulation are included (upper trace: wt; bottom trace: Npc1^nmf164^). Scale bar: −1 mV/5 ms. **C** mGluR-LTD expressed as excitatory postsynaptic field responses (fEPSP) registered in the CA1 pyramidal region of wt and Npc1^nmf164^ mice before and after 10 min of incubation with DHPG (non-membrane permeable mGluR_5_ agonist). Graphs represent fEPSP slope over baseline (left) or mGluR-LTD expressed as mean ± SEM percentage of fEPSP slope over baseline from minute 45 to minute 55 of the LTD recording (right) (*P*_*WT*_ < 0.0001; *n* = 7–9; Student’s *t* test*;* # comparison of baseline *versus* LTD for the same genotype; *comparison between different genotypes). Representative traces of responses to stimulation are included (upper trace: wt; bottom trace: Npc1^nmf164^). Scale bar: −1 mV/5 ms. **D** mGluR-LTD expressed as excitatory postsynaptic field responses (fEPSP) registered in the CA1 pyramidal region of wt and Npc1^nmf164^ mice before and after 15 min of incubation with CHPG (membrane-permeable mGluR_5_ agonist). Graphs represent fEPSP slope over baseline (left) or mGluR-LTD expressed as mean ± SEM percentage of fEPSP slope over baseline from minute 55 to minute 65 of the LTD recording (right) (*P*_*Npc1*_ = 0.0004; *P*_*WT-Npc1*_ = 0.0425; *n* = 5-10; Student’s *t* test, # comparison of baseline *versus* LTD for the same genotype; *comparison between different genotypes). Representative traces of responses to stimulation are included (upper trace: WT; bottom trace: Npc1^nmf164^). Scale bar: −1 mV/5 ms.

We next aimed to determine whether increased mGluR_5_ levels upon NPC1 deficiency resulted in enhanced mGluR_5_ function. As mGluR_5_ activity requires protein synthesis [19], we employed the SUnSET assay, which allows for the detection of newly synthesized proteins by measuring puromycin incorporation [20, 21]. We observed a 59% increase in basal protein synthesis in hippocampal slices from Npc1^nmf164^ mice compared with wt mice (Fig. 3A). To monitor synaptic plasticity in response to specific mGluR_5_ activation, we performed electrophysiological analysis of mGluR-LTD in hippocampal slices of wt and Npc1^nmf164^ mice after synaptic stimulation (paired pulses delivered at low frequency, in the presence of the NMDA receptor antagonist AP5). LTD was significantly induced in both wt and Npc1^nmf164^ slices. However, mGluR-LTD expression in Npc1^nmf164^ slices was stronger than in the wt slices, in agreement with the increased mGluR_5_ levels (Fig. 3B). To differentiate between cell surface and intracellular mGluR_5_ function, either the non-membrane-permeable (S)-3,5-dihydroxyphenylglycine (DHPG) [22, 23] or the membrane-permeable (R,S)-2-chloro-5-hydroxyphenylglycine (CHPG) [24] chemical agonists of mGluR_5_ were used in wt and Npc1^nmf164^ slices. When compared to baseline internally by genotype, LTD was significantly induced in wt slices in response to DHPG but not CHPG, while in the Npc1^nmf164^ slices LTD expression was observed upon incubation with CHPG but not DHPG. These results are consistent with the higher mGluR5 expression in the surface in wt or intracellular in Npc1^nmf164^ conditions. When LTD expression was compared between genotypes, the Npc1^nmf164^ slices expressed 14.8% less LTD in response to DHPG (Fig. 3C) and 137% more (Fig. 3D) in response to CHPG, than the wt slices. This is also in agreement with reduced surface and increased intracellular mGluR_5_ levels in Npc1^nmf164^ conditions.

### Oral treatment with the mGluR_5_ antagonist CTEP normalizes mGluR-LTD and ameliorates behavioral alterations in Npc1^nmf164^ mice

**Fig. 4:**
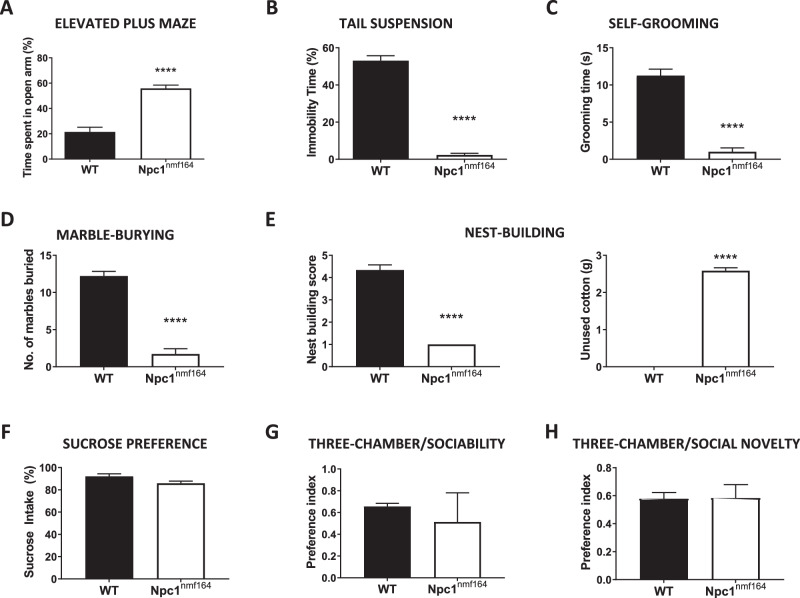
Behavioral alterations in Npc1^nmf164^ mice. **A** Graph showing mean ± SEM time spent in the open arm of the elevated plus maze as a percentage of the total exploring time by wt or Npc1^nmf164^ mice (*P* < 0.0001; *n* = 9-10; Student’s *t* test). **B** Graph showing mean ± SEM immobility time spent in the tail suspension test as a percentage of the total time by wt or Npc1^nmf164^ mice (*P* < 0.0001; *n* = 9-11; Student’s *t* test). **C** Graph showing mean ± SEM of time spent self-grooming by wt or Npc1^nmf164^ mice (*P* < 0.0001; *n* = 7–8; Student’s *t* test). **D** Graph showing mean ± SEM of number of marbles buried by wt or Npc1^nmf164^ mice (*P* < 0.0001; *n* = 7–9; Student’s *t* test). **E** Graphs show mean ± SEM of nest-building test score (left) and unused cotton material in grams (right) by wt or Npc1^nmf164^ mice in the nest-building test (*P*_*left*_ < 0.0001; *P*_*right*_ < 0.0001 n = 7-9; Student’s *t* test). **F** Graph showing mean ± SEM of sucrose solution consumption in the sucrose preference test by wt or Npc1^nmf164^ mice (*n* = 4; Student’s *t* test). **G** Graph showing mean ± SEM of preference index for sociability (stranger 1 vs. empty cage) in the three-chamber test by wt or Npc1^nmf164^ mice (*n* = 3–4; Student’s *t* test). **H** Graph showing mean ± SEM Graph shows mean ± SEM of preference index for social novelty (stranger 2 vs. stranger 1) in the three-chamber test by wt or Npc1^nmf164^ mice (*n* = 3–4; Student’s *t* test).

**Fig. 5:**
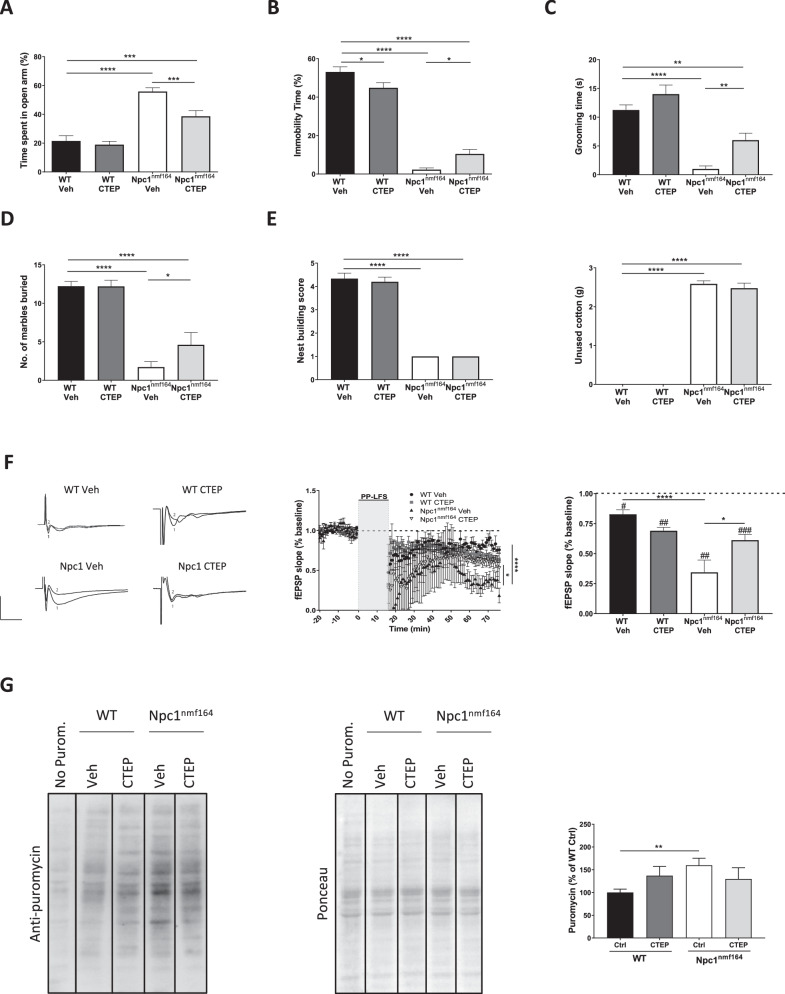
The mGluR_5_ antagonist CTEP normalizes mGluR_5_ function and psychiatric alterations in Npc1^nmf164^ mice. **A** Graph showing mean ± SEM time spent in the open arm of the elevated plus maze as a percentage of the total exploring time by wt or Npc1^nmf164^ mice treated with vehicle (Veh) (data from Fig. 4 are represented here in (**A**–**E**) for clarity) or with CTEP (membrane-permeable mGluR_5_ antagonist) (*P*_*WT Veh – Npc1 Veh*_ < 0.0001; *P*_*Npc1 Veh – Npc1 CTEP*_ = 0.0006; *P*_*WT Veh – Npc1 CTEP*_ = 0.0008; *n* = 9–10; grouped one-way ANOVA Bonferroni post hoc). **B** Graph showing mean ± SEM immobility time spent in the tail suspension test as a percentage of the total time by wt or Npc1^nmf164^ mice treated with Veh or CTEP (*P*_*WT Veh – WT CTEP*_ = 0.0169; *P*_*WT Veh – Npc1 Veh*_ < 0.0001; *P*_*Npc1 Veh – Npc1 CTEP*_ = 0.0111; *P*_*WT Veh – Npc1 CTEP*_ < 0.0001; *n* = 9–11; grouped one-way ANOVA Bonferroni post hoc). **C** Graph showing mean ± SEM of time spent self-grooming by wt or Npc1^nmf164^ mice treated with Veh or CTEP (*P*_*WT Veh – Npc1 Veh*_ < 0.0001; *P*_*Npc1 Veh – Npc1 CTEP*_ = 0.002; *P*_*WT Veh – Npc1 CTEP*_ = 0.001; *n* = 5–8; grouped one-way ANOVA Bonferroni post hoc). **D** Graph showing mean ± SEM of number of marbles buried by wt or Npc1^nmf164^ mice treated with Veh or CTEP (*P*_*WT Veh – Npc1 Veh*_ < 0.0001; *P*_*Npc1 Veh – Npc1 CTEP*_ = 0.0407; *P*_*WT Veh – Npc1 CTEP*_ < 0.0001; *n* = 5–9; grouped one-way ANOVA Bonferroni post hoc). **E** Graphs showing mean ± SEM of nest-building test score (left) and unused cotton material in the nest-building test (right) by wt or Npc1^nmf164^ mice treated with Veh or CTEP (*P*_*Left WT Veh – Npc1 Veh*_ < 0.0001; *P*_*Left WT Veh – Npc1 CTEP*_ < 0.0001; *P*_*Right WT Veh – Npc1 Veh*_ < 0.0001; *P*_*Right WT Veh – Npc1 CTEP*_ < 0.0001; *n* = 5–9; Grouped one-way ANOVA Bonferroni post hoc). **F** mGluR-LTD expressed as excitatory postsynaptic field responses (fEPSP) registered in the CA1 pyramidal region of wt and Npc1^nmf164^ mice treated with CTEP or Veh. Graphs represent fEPSP slope over baseline (left) or mGluR-LTD expressed as mean ± SEM percentage of fEPSP slope over baseline from minute 45 to minute 55 of the LTD recording (right) (*P*_*WT Veh*_ = 0.0110; *P*_*WT CTEP*_ = 0.0087; *P*_*Npc1 Veh*_ = 0.0080; *P*_*Npc1 CTEP*_ = 0.0003; *P*_*WT-Npc1*_ < 0.0001; *P*_Npc1 Veh – Npc1 CTEP_ = 0.0136; *n* = 5 slices from 5 wt mice treated with CTEP or Veh and *n* = 5 slices from 5 Npc1^nmf164^ mice treated CTEP or Veh; # comparison of baseline *versus* LTD for the same genotype; *comparison between different genotypes; grouped one-way ANOVA Bonferroni post hoc). Representative traces of responses to stimulation are included (upper left trace: wt Veh; upper right trace: wt CTEP; bottom left trace: Npc1^nmf164^ Veh; bottom right trace: Npc1^nmf164^ CTEP). Scale bar: −1 mV/5 ms. **G** Western blot showing puromycin in the Sunset assay in slices from the hippocampus from wt and Npc1^nmf164^ mice treated with CTEP or Veh. Graph shows mean ± SEM total intensity associated with puromycin as a percentage of the values obtained in wt slices treated with Veh (*P* = 0.0051; *n* = 9–18; grouped one-way ANOVA Bonferroni post hoc).

Altered mGluR_5_ function has been linked to psychiatric conditions [11] that often affect patients with NPC, especially those affected by late-onset forms of the disease. The Npc1^nmf164^ mouse model is considered a better model for late-onset NPC than the Npc1 null mouse, and develops disease symptoms in a wider time window allowing behavioral assessment. While motor and cognitive impairments have been characterized in the Npc1^nmf164^ mice [8, 9, 25], little is known about the psychiatric alterations in this NPC mouse model [25]. Thus, a series of behavioral tests were conducted at 3.5-months-of-age. Npc1^nmf164^ mice showed remarkable alterations in the elevated plus maze test related to anxiety (Fig. 4A), the tail suspension test related to depressive state (Fig. 4B), the self-grooming test commonly used to assess stereotypic behaviors (Fig. 4C), the marble burying test related to obsessive-compulsive behavior (Fig. 4D) and the nest building test to assess a natural home-caged behavior (Fig. 4E). This aberrant conduct was already evident in Npc1^nmf164^ mice at 2.5 months of age (Supplementary Fig. S8), the time when altered mGluR5 levels and localization were described (see Fig. 1). In contrast, Npc1^nmf164^ mice did not show anomalous behavior in the sucrose preference test related to anhedonia (Fig. 4F), or in tests aimed at measuring sociability (Fig. 4G) and social memory (Fig. 4H). To determine whether mGluR_5_ enhancement could be related to the anomalous behaviors, we treated 3-month-old Npc1^nmf164^ mice with the membrane permeable mGluR_5_ antagonist CTEP [26]. Since this allosteric modulator crosses the brain-blood barrier, it was administered by oral gavage every 2 days for a total of 15 days. CTEP treatment improved Npc1^nmf164^ mouse behavior in the elevated plus maze, tail suspension, self-grooming and marble burying tests (Fig. 5A, B, C, D) but did not affect the altered behavior of Npc1^nmf164^ mice in the nest building test (Fig. 5E). CTEP treatment did not have any significant effect in wt mice with the exception of increased immobility in the tail suspension test (Fig. 5A–E). CTEP did not improve the impaired memory of Npc1^nmf164^ mice as evidenced by the Y maze test (Supplementary Fig. S9). Electrophysiological measurement in hippocampal slices obtained from the wt or Npc1^nmf164^ mice after the behavioral assessment confirmed that CTEP treatment prevented the enhanced mGluR-LTD in the Npc1^nmf164^ mice (Fig. 5F). CTEP also showed a tendency to reduce the abnormally increased protein synthesis in Npc1^nmf164^ mice, as evidenced by the SUnSET assay, although the reduction did not reach statistical significance (Fig. 5G).

## Discussion

Patients with NPC, especially those affected by late-onset forms of the disease, suffer a variety of psychiatric manifestations that may include behavioral alterations, psychosis and dementia [27–29]. The psychiatric symptomatology not only affects deeply the quality of life of patients and their families but also confounds NPC diagnosis [2, 3, 14]. NPC patients are often misdiagnosed for more prevalent diseases such as obsessive-compulsive or autism-like disorders [30]. Thus, it is urgent to understand and treat the NPC psychiatric condition. Here, we have performed a detailed characterization of mood anomalies in the Npc1^nmf164^ mice, which develop disease more slowly than NPC1-null mice [8]. We found remarkable alterations in the elevated plus maze and tail suspension tests, which could be interpreted as reduced anxiety and depressive state. However, these results could also be seen as difficulty to evaluate risks and lack of impulse control that match behavior reported in patients [30] and, together with stereotypic behavior and impairment of memory and learning [9], validate Npc1^nmf164^ mice as animal model for the study of neurological NPC.

The results we have obtained in the Npc1^nmf164^ mice and in Npc1 deficient cultured neurons, together with the observations made on the brain tissue of a patient with NPC, point for the first time to alterations on mGluR_5_ levels and function as a relevant cause for the psychiatric condition in NPC. Previous work performed in NPC1 null cortical neurons reported alterations in mGluR_5_. In particular, the presence of the receptor was found reduced in cholesterol-enriched lipid rafts at the plasma membrane, and stimulation of mGluR_5_ by the non-membrane permeable agonist DHPG improved the defective internalization of AMPA receptors observed in these neurons [31]. These findings led the authors to propose that stimulation of mGluR_5_ could be a therapeutic strategy for NPC. Here, we confirmed the reduction of mGluR_5_ at the plasma membrane in Npc1^nmf164^ neurons. However, we also unveiled the accumulation of the receptor in the endolysosomal compartment resulting in increased overall mGluR_5_ activity. Hence, our findings not only raise concerns about the use of mGluR_5_ stimulation in the NPC clinical context but support its intracellular inhibition as a promising therapeutic approach.

In an effort to characterize the molecular mechanisms underlying the mGluR_5_ phenotype, we analyzed its gene expression, and the presence of the receptor in lipid raft domains. Similar mGluR_5_ mRNA levels were found in the hippocampus of Npc1^nmf164^ and wt mice, which argues against increased receptor synthesis but in favor of impaired protein degradation as an explanation for its unusually high levels. We did not observe significant alterations in mGluR_5_ raft partitioning in Npc1^nmf164^ neurons, unlike the reduction observed in NPC1-null neurons [31]. This finding argues against an altered internalization of mGluR_5_, for which raft domains are crucial [13], as a reason for receptor misdistribution. Instead, it is likely that the impaired lysosomal function due to cholesterol accumulation traps mGluR_5_ in these organelles where it cannot be degraded efficiently. Accumulation of several other proteins such as TLR4 in the endolysosomal compartment of NPC cells have been described [32]. In the case of TLR4 it was proposed that the protein is overactivated in endosomes and, due to impaired membrane flow, it cannot be properly sorted to lysosomes where its activity would be turned off. Aberrant endosomal accumulation and activation of TLR4 would lead to increased cytokine secretion in NPC fibroblasts and glial cells [32]. We here propose that mGluR_5_ reaches the lysosome where it can be active. The importance of lysosomal dysfunction in the NPC1 mGluR_5_ phenotype is supported by experiments where lysosomal function was inhibited with bafilomycin in wt neurons and by the unaltered mGluR_5_ expression in other intracellular organelles in Npc1^nmf164^ neurons. The electrophysiology experiments using non-permeable and permeable mGluR_5_ agonists, together with the increased basal translation observed in the Npc1^nmf164^ hippocampal slices, indicate that the mGluR_5_ accumulating in the endolysosomal compartment remains active leading to increased mGluR_5_ dependent LTD despite a decrease in receptor levels at the neuronal surface. While the presence of mGluR_5_ had been described in lysosomes [33], this pool was not considered active until recent work demonstrated its functionality in glial cells [34]. Studies performed in the striatum [12] and in the hippocampus [24] have shown that activation of intracellular mGluR5 up-regulates genes involved in synaptic plasticity including activity-regulated cytoskeletal-associated protein (Arc/Arg3.1), which would sustain LTD. The intracellular availability of glutamate [35] could ensure activation of the endolysosomal mGluR_5_ [35], which would have the right topology to trigger LTD signaling pathways in the cytosol.

Interestingly, neurons seem more prone to show mGluR_5_ intracellular accumulation than astrocytes, a cell type where mGluR_5_ is the most abundant metabotropic receptor and plays a relevant role in the calcium regulation [36]. Recent studies have shown variable mGluR_5_ expression over time in cortical astrocytes, which may undergo transient increases to regulate synaptic transmission [37]. While we have not found significant changes in mGluR_5_ expression in astrocytes from the Npc1^nmf164^ mouse cortex at 2 months of age, we cannot rule out transient changes or an increase at later stages of the disease.

NPC is a fatal disease. The iminosugar miglustat, which by inhibiting the glucosylceramide synthase reduces ganglioside accumulation, is the only approved treatment that delays neurological symptoms but has side effects [38]. Our findings contribute to the understanding of the NPC psychiatric condition, and provide a potential new therapeutic strategy based on the membrane-permeable mGluR_5_ antagonist CTEP. Rather than directly targeting lipid build-up, CTEP counteracts the aberrant increase in the activity of intracellular mGluR_5_. Since mGluR_5_ function triggers protein synthesis [19], the aberrant overactivation of this receptor in NPC may be one of the causes contributing to the protein synthesis upregulation we observed in NPC1 hippocampal slices using the SunSET assays. However, dysregulation of other signaling pathways reported in NPC cells such as PI3K/Akt-GSK-3β-NF-κB [39] or mTORC1 [40], would contribute as well and may explain why the reduction on protein synthesis observed after mGluR_5_ inhibition does not reach statistical significance. On the other hand, although CTEP treatment did not revert impaired hippocampal memory, it significantly improved the alterations related to anxiety and depressive state in Npc1^nmf164^ mice, as well as those related to obsessive-compulsive and stereotypic behavior. Benefits were observed even when treatment started at advanced stages of the disease, after the onset of the psychiatric condition. This is important for its potential application to patients with NPC, considering the frequent delay in clinical diagnosis. The ability of CTEP to cross the blood-brain barrier allows oral administration, which also eases its clinical application. Preclinical work has shown the beneficial effects of CTEP in mouse models for other more common neurodegenerative diseases such as Alzheimer’s [41], Huntington’s [42], Parkinson’s [43] or Fragile-X [44]. Allosteric negative modulation of mGluR_5_ has also been assessed to treat inflammatory pain [45] and migraine [46]. Acquired treatment resistance may occur following chronic mGluR_5_ inhibition, but it is not inevitable if the pharmacological interventions are timed in critical windows [44]. Efforts are ongoing to improve mGluR_5_ inhibitory strategies and to generate novel allosteric modulators. Here, we provide proof-of-concept for their general suitability for the treatment of NPC.

Cholesterol accumulation in lysosomes resulting in impaired function of these organelles characterizes other lysosomal storage disorders in which psychiatric condition is a hallmark. We propose that this could result in increased intracellular mGluR_5_ activity and that its inhibition by membrane-permeable antagonists might be a common therapeutic strategy to improve the day-to-day life of patients suffering from these devastating diseases.

## Materials and methods

### Mice

C57BL/6J-Npc1^nmf164/J^ heterozygous mice [8] were purchased from Jackson laboratories and the mouse colony established and kept in specific-pathogen-free housing at the Centro Biología Molecular Severo Ochoa (CBM). PCR was used to genotype the male and female wt and homozygous Npc1^nmf164^ mice as in [9]. Mice experimental groups, including similar numbers of males and females, were used at 2.5months of age for the analysis of mGluR5 levels and distribution (*n* = 4–6) and at 2.5 and 3.5 months of age for the behavioral assessment (*n* = 5-11). Results showed no gender-dependent differences in any instance.

### Human samples

Formaldehyde-fixed brain tissue from a control child and an NPC patient child were donated by the Fundación CIEN and the Vall d’Hebron Hospital, respectively.

### Antibodies

We used antibodies against: anti-nuclear pore complex (#MMS-120P, Covance); anti-calnexin (#ab219644, Abcam); anti-caveolin1 (#ab36152, Abcam); anti-complex IV (#1D6E1A8, Invitrogen); anti-EEA1 (#48453, Cell Signalling); anti F4/80 (#6640, Abcam); anti-flotilin1 (#610821, BD Bioscience); anti-GFAP (#MAB3402, Merck); anti-GM130 (#610823, BD Bioscience); anti-Homer1 (#160004, Synaptic Systems); anti-Lamp1 (#1D4B, DSHB); anti-MAP2 (#822501, Biolegend); anti-mGluR_5_ (#AB5675, Millipore); anti-mGluR_5_ N-term (#AGC-007, Alomone Labs); anti-mGlu_5_ C-term (#MSFR104140, mGluR_5_-Rb-Af300; Nittobo Medical Co. Ltd., Hokkaido, Japan); anti-NPC1 (#nb400-148ss); anti-puromycin (#PMY-2A4, DSHB); anti-tGFP (#TA150041, Origene); anti-tubulin (#T5168, Sigma).

### Neuronal cultures

Primary cultures of neurons were obtained from the hippocampus of day 18 mouse embryos following the protocol established in [47]. We used Neurobasal medium (#21103–049, Thermo Fisher Scientific,) supplemented with B27 (#17504044, Thermo Fisher Scientific) and GlutaMAX (#35050061, Thermo Fisher Scientific) to culture the neurons at 5% CO_2_ and 37 °C. GlutaMAX was removed from the medium at day 7 in vitro as indicated in [9]. Experiments were performed at day 14 in vitro.

### In vitro treatments

Were performed in neuronal cultures obtained from wt mouse embryos. Where indicated, U18666A (#B6BC5106, Sigma) 2 µg/µl in dimethyl sulfoxide (DMSO), 2-hydroxypropyl-β-cyclodextrin (#H107, Sigma) 100 µM in H_2_O or bafilomycin A1 (#BML-CM110-0100, Enzo) 0,1 µM in H_2_O, were added to the medium for 24 h.

### Generation of lentiviral vectors

Production of lentiviruses was done in HEK 293-T cell line with P2 biosecurity level as described in [48]. Neuronal cultures were infected at 6 DIV with a multiplicity of infection = 5. The next day, the medium was completely replaced by B27-supplemented Neurobasal medium.

### Analysis of protein expression by western blot

Brain or cellular extracts were prepared in 2-morpholinoethanesulfonic acid (MES) pH 7, ethylenediaminetetraacetic acid (EDTA) 2 mM with protease inhibitors (cOmplete^TM^, Sigma-Aldrich) and phosphatase inhibitors (#P5726, Sigma Aldrich). Homogenates were separated by electrophoresis, transferred onto nitrocellulose membranes and incubated with primary and secondary antibodies using conventional protocols. Proteins were detected with luminol (Pierce^TM^ ECL Western Blotting Substrate, Thermo Fisher Scientific) and chemiluminescence was measured using a CCD camera (Amersham Imager 680) and the levels quantified by the FIJI image-processing software.

### Quantitative RT–PCR

Total RNA was extracted from brain tissue from wt and Npc1^nmf164^ mice using TRIzol Reagent (Ambion/RNA Life Technologies Co.) qRT-PCR was performed as in [9] using the following primers purchased from Sigma-Aldrich (mouse mGluR5: forward: 5′-CTTAGATCGCAGCCACTAGC-3′ and reverse 5′-GTAAAATCACCAGGTGCGCT-3′). Three housekeeping genes (Gapdh, GusB, and Pgk1) were used as endogenous controls.

### Immunofluorescence analysis (IFA)

Cultured neurons at 14 DIV were fixed in 4% paraformaldehyde (PFA) with 0.12 M sucrose, permeabilized with 0.1% Triton X-100 and incubated overnight with primary antibodies and subsequently with Alexa conjugated secondary antibodies. For surface staining of mGluR_5_, cultured neurons at 14 DIV were fixed in 4% PFA with 0.12 M sucrose and blocked in 2% bovine seroalbumin without detergent to avoid permeabilization. The fixed neurons were incubated overnight with a primary antibody (#AGC-007, Alomone Labs) that specifically recognizes mGluR_5_ N-terminal domain, which is exposed extracellularly. Only afterwards, cells were permeabilized with 0.1% Triton X-100 and incubated with DAPI or other antibodies to access intracellular compartments.

For immunofluorescence in mouse brains, these were fixed in 4% PFA 0.12 M sucrose and cryoprotected as indicated in [9]. Sagittal sections of 30 µm were obtained and incubated with the primary antibodies (overnight at 4 °C) and with Alexa-conjugated secondary antibodies. A 10 min incubation with DAPI (Merck) was performed to stain cell nuclei. Sections were mounted with Mowiol-Dabco (Mowiol, Chalbiochem).

For immunofluorescence in human tissue, sections were deparaffinized in decreasing concentrations of ethanol and xylene. Heat-mediated antigen retrieval was conducted in Tris–EDTA buffer (pH 9.0). The sections were processed similarly to the mouse brain sections and mounted using FluorSave (Calbiochem).

The images were obtained in all cases using a Zeiss confocal microscope LSM710 and quantified by Fiji software. The JACoP plugin was applied to obtain Manders’ overlap coefficient [49].

### Electron microscopy and immunogold labeling

Npc1^nmf164^ mice and wt littermates were intracardially perfused with phosphate buffer saline (PBS) and fixative (4% PFA and 0.05% glutaraldehyde in 0.1 M PB pH 7.4) as described in [50]. Blocking of 60-μm-thick sections, incubation with affinity-purified polyclonal anti-mGluR_5_ antibody and with goat anti-rabbit IgG coupled to 1.4 nm gold, and processing for electron microscopy were performed as in [50]. Ultrathin sections (60–90 nm) were obtained using an ultramicrotome (Leica Ultracut UCT) and visualized on a Jeol-1010 electron microscope (Jeol, Tokyo, Japan). Images were obtained with a Galan digital camera (Erlangshem ES1000W Model. 785).

### DRM isolation

Neuronal extracts containing 200μg protein were incubated in TNE buffer (Tris-HCl 50 mM pH 7.4, NaCl 150 mM y EDTA 5 mM) containing 1% Triton X-100 and protease inhibitors at 4 °C for 40 min under rotation. Samples were then centrifuged at 100,000 *g* for 1 h at 4 °C. The pellet was considered the detergent-resistant fraction (DRM).

### Lyosomal isolation from mouse brain

Subcellular fractionation and isolation of a lysosomal-enriched fraction from wt and Npc1^nmf164^ mice brains were performed using a protocol inspired in [51, 52]. Briefly, mice were sacrificed by cervical dislocation and their brains were extracted and resuspended in 0.25 M sucrose. For each fractionation protocol two mice brains per experimental group were pooled. Isolation was achieved through a series of centrifugations including a nycodenz-density-gradient centrifugation (50, 26, 24, 20, and 15%) recovering the lysosomal fraction at the 24–26% interface. The organelles were washed in 0.25 M sucrose, centrifuged and the pellets were resuspended in water (with protease and lipase inhibitors) and stored at −80 °C.

### Cholesterol visualization

Cultured neurons were incubated with the fluorescent tracer BODIPY-cholesterol (Top Fluor Cholesterol/23-(dipyrrometheneboron difluoride)-24-norcholesterol (#878557-19-8, Avanti Polar Lipids, Inc.)), which was dissolved in DMSO and added to the cell medium at a final concentration of 1 μM for 24 h as in [53]. Fluorescent images of BODIPY-cholesterol and of Lamp1 signal obtained by immunofluorescence were taken using a confocal LSM710 vertical microscope. For quantification, and to capture the difference between lysosomal and cytosolic signal without overlap, we applied a threshold to select the Lamp1 positive area and a second threshold to select the cytosolic region of each cell. The raw values of fluorescence were normalized using the area of each selected region.

### Protein synthesis: SUnSET

The non-radioactive method known as SUnSET (SUrface Sensing of Translation) was used for protein synthesis quantification as indicated in [20, 21].

### Electrophysiological recordings

Mice hippocampal slice preparation and recording of field excitatory postsynaptic potentials (fEPSPs) was performed as indicated in [9]. LTD was induced by pair-pulse low-frequency stimulation (PP-LFS) of Schaffer collateral fibers (900 pair-pulses separated by 50 ms at 1 Hz). Responses were recorded for 1 h after induction. pClamp9 software (Molecular Devices) was used for acquisition. For the chemical-induced LTD, 100 µM DHPG (#0342, Tocris Bioscience) or 100 µM CHPG (#1049, Tocris Bioscience) was added to the artificial cerebrospinal fluid (aCSF) for 10 or 15 min, respectively, and responses were recorded up to 1 h after induction.

### In vivo treatments

CTEP (RO4956371, #V1084, InvivoChem) was dissolved in vehicle (Veh: NaCl 0,9% and Tween-80 0.3%) and administered to the mice by oral gavage at 2 mg/kg every 48 h. As a control, wt and Npc1^nmf164^ mice received the same volume of Veh. The chronic study was initiated at 14 weeks of age and prolonged for another 2 weeks.

### Behavioral tests

The elevated plus maze was performed as described in [54]. The tail suspension test was performed as described in [55]. The self-grooming and marble burying tests were performed as described in [20]. The nest building test was performed as described in [56].

The sucrose preference test was performed as described in [57]. The sociability and social novelty tests were evaluated using the three-chamber test as described in [20]. The Y-maze test was performed as described in [58].

### Statistical analysis

The number of mice used was selected on the basis of previous phenotyping analyses conducted in the same model and calculating the statistical power of the experiment. Mice were genotyped and according to the genotype randomly assigned to the experimental groups. No outliers were excluded in the study. The information about sample collection, treatment and processing is included in Results and Material and Methods sections. Investigators assessing and measuring results were blinded to the intervention. All statistical comparisons were based on biological replicates. Data are presented as the mean ± SEM. We assessed data normality by the Shapiro–Wilk test. For two-group comparisons, we used two-sample Student’s *t* test for data with parametric distribution. For multiple comparisons, we used one-way or two-way ANOVA followed by Bonferroni *post hoc* test for data with normal distribution. *P*-values (*P*) ≤ 0.05 were considered significant and indicated in the figures by hash when comparing the same genotype, or by asterisk when comparing different genotypes: ^#^*≤ 0.05; ^##^**≤ 0.01; ^###^***≤ 0.001. We used GraphPad Prism 7.0 software (GraphPad Software, La Jolla, CA, USA) for all statistical analysis.

## Supplementary information

Supplemental material Figures S1-S9

Figure S10, Uncropped blots

## Data Availability

The experimental data sets generated and/or analyzed during the current study are available from the corresponding author upon reasonable request. No applicable resources were generated during the current study.
