# Supplementary material for: Enhanced mGluR5 intracellular activity causes psychiatric alterations in Niemann Pick type C disease
Source: Cell Death Dis. 2024 Oct 23;15(10):771. doi: 10.1038/s41419-024-07158-8 (PMC11499878; doi:10.1038/s41419-024-07158-8)
Supplement: Supplementary file 1 — Supplemental material Figures S1-S9 [file 41419_2024_7158_MOESM1_ESM.pdf]

SUPPLEMENTARY MATERIAL

Enhanced $mGluR_5$ intracellular activity causes psychiatric alterations in Niemann Pick type C disease

Ana Toledano-Zaragoza, Violeta Enriquez-Zarranlaga, Sara Naya-Forcano, Víctor Briz, Rocío Alfaro-Ruíz, Miguel Parra-Martínez, Daniel N. Mitroi, Rafael Luján, José A. Esteban, María Dolores Ledesma

Table of content

Figure S1. mGluR5 mRNA levels in the brain of wt and $Npc1^{nmf164}$ mice

Figure S2. Isolation of a lysosomal-enriched fraction from wt and $Npc1^{nmf164}$ mice

Figure S3. mGluR5 alterations in genetically NPC1-silenced cultured neurons

Figure S4. mGluR5 alterations in primary cultured neurons derived from $Npc1^{nmf164}$ mice

Figure S5. mGluR5 subcellular distribution in wt and NPC1-deficient cultured neurons

Figure S6. mGluR5 levels in wt and NPC1-deficient cultured astrocytes

Figure S7. mGluR5 distribution after lysosomal inhibition

Figure S8. Behavioural alterations in $Npc1^{nmf164}$ mice at 2.5 months of age

Figure S9. CTEP treatment does not ameliorate memory deficits in $Npc1^{nmf164}$ mice

FIGURE S1

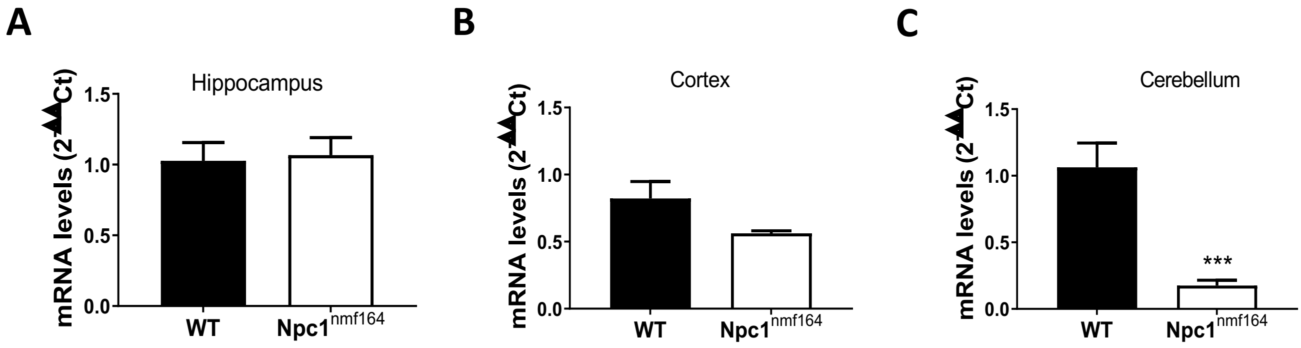

Figure S1. mGluR5 mRNA levels in the brain of wt and Npc1^{nmf164} mice

Graph showing mean ± SEM levels of mGluR$_5$ mRNA quantified by qRT-PCR in the hippocampus (a), cortex (b) and cerebellum (c) of 10 week-old wt or Npc1^{nmf164} mice ($P_{cerebellum}$ = 0.0002; n = 4-7; Student's *t-test*).

FIGURE S2

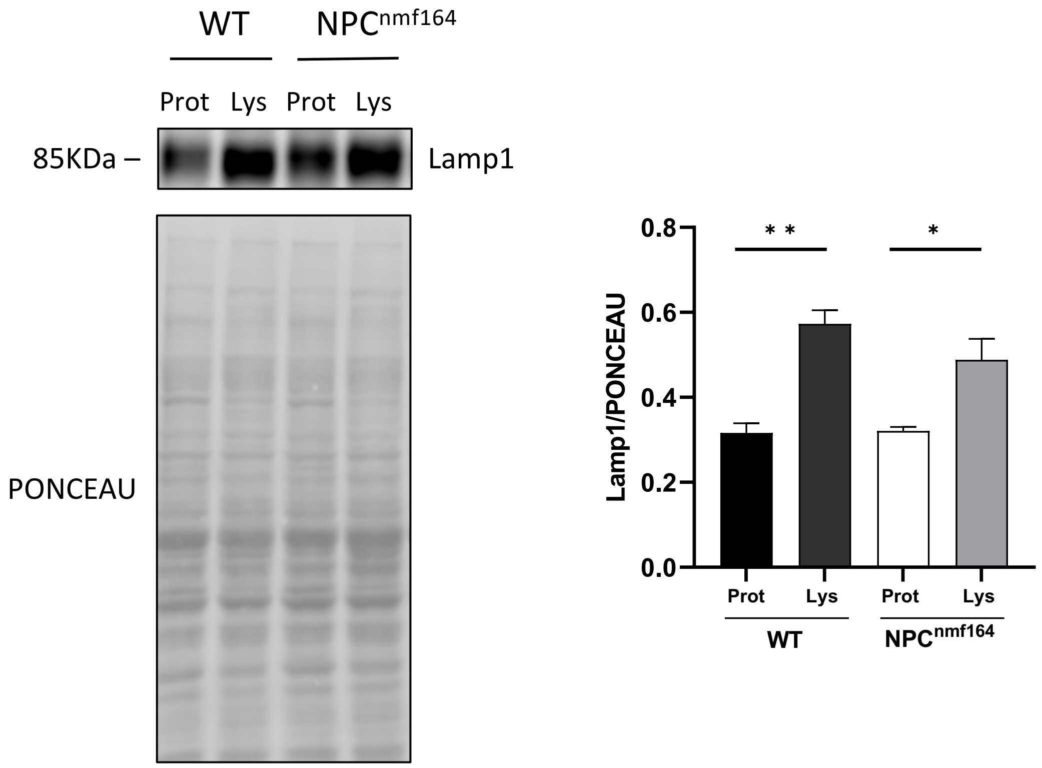

Figure S2. Isolation of lysosomal-enriched fractions from the brain of wt and Npc1^{nmf164} mice

Western blot analysis of the lysosomal marker Lamp1 in the total protein extract, which served as starting fraction of the isolation protocol (Prot), and in the fraction corresponding to the interface 24-26% nycodenz gradient in which lysosomes are recovered (Lys). Lower panel shows Ponceau staining confirming similar amount of protein loaded in each lane. Graph shows mean ± SEM levels of Lamp1 in each fraction normalized to the ponceau staining in wt and Npc1^{nmf164} mice (P_{WT} = 0.0027 ; $P_{NPC}{}^{nmf164}$ = 0.0293); n = 3 independent isolations using two brains in each of them; Paired T-test).

FIGURE S3

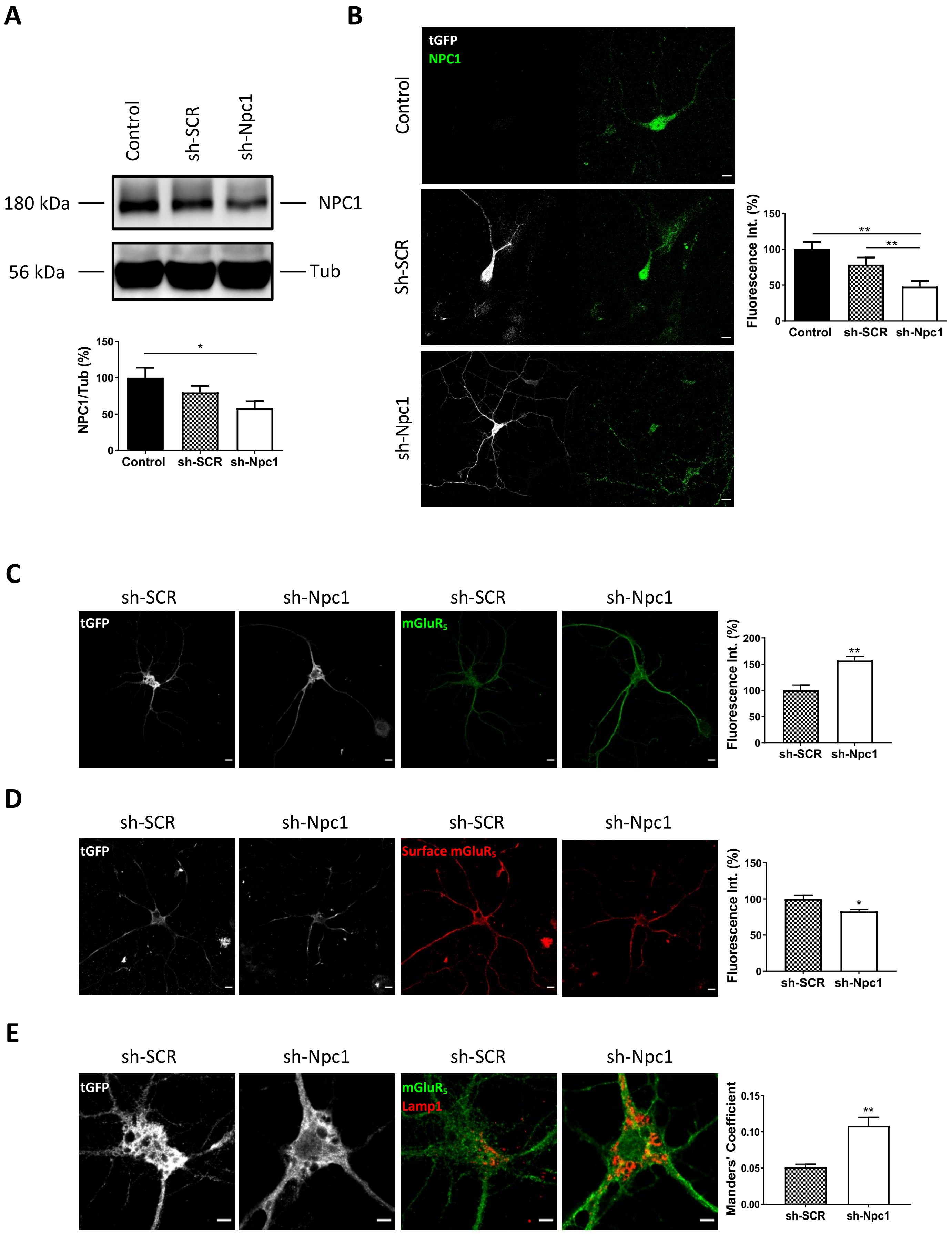
A
Control
sh-SCR
sh-Npc1
180 kDa
NPC1
56 kDa
Tub
NPC1/Tub (%)
150
100
50
0
*
Control
sh-SCR
sh-Npc1
B
tGFP
NPC1
Control
Sh-SCR
sh-Npc1
Fluorescence Int. (%)
150
100
50
0
**
**
Control
sh-SCR
sh-Npc1
C
sh-SCR
sh-Npc1
sh-SCR
sh-Npc1
tGFP
mGluR5
Fluorescence Int. (%)
200
150
100
50
0
**
sh-SCR
sh-Npc1
D
sh-SCR
sh-Npc1
sh-SCR
sh-Npc1
tGFP
Surface mGluR5
Fluorescence Int. (%)
150
100
50
0
*
sh-SCR
sh-Npc1
E
sh-SCR
sh-Npc1
sh-SCR
sh-Npc1
tGFP
mGluR5
Lamp1
Manders' Coefficient
0.15
0.10
0.05
0.00
**
sh-SCR
sh-Npc1

Figure S3. mGluR5 alterations in genetically NPC1-silenced cultured neurons

A. Western blot showing NPC1 and tubulin (Tub) in cultured neurons from wt mice incubated with vehicle (Veh) or with lentiviruses encoding sh-RNA specific for Npc1 (sh-Npc1) or scrambled control (sh-SCR). Graph shows mean ± SEM levels of NPC1 normalized to Tub as a percentage of the values obtained in the Veh-incubated neurons ($P = 0.0354$; n = 6; grouped one-way ANOVA Bonferroni *post hoc*).

B. Immunofluorescence analysis (IFA) of NPC1 and tGFP, which identifies transfected neurons, in cultured neurons from wt mice incubated with Veh or with lentiviruses encoding sh-SCR or sh-Npc1. Graph shows NPC1 associated intensity as a percentage of the values obtained in the Veh- incubated neurons ($P_{control\text{-}shNPC1} = 0.0084$; $P_{shSCR\text{-}shNPC1} = 0.0093$; n = 6; grouped one-way ANOVA Bonferroni *post hoc*). Scale bar 10 µm.

C.D. IFA of total (c) and surface (d) $mGluR_5$ in cultured neurons transfected with either sh-SCR or sh-Npc1 RNA as indicated by the tGFP positive signals. Graphs shows mean ± SEM fluorescence intensity associated with $mGluR_5$ as a percentage of the values obtained in the sh-SCR transfected neurons ($P_C = 0.009$; $P_D = 0.0304$ n = 3-4; paired Student's *t*-test). Scale bar 10 µm.

E. IFA of $mGluR_5$ and Lamp1 (endolysosomal marker) in cultured neurons transfected with either sh-SCR or sh-NPC1 RNA as indicated by the tGFP positive signals. Graph shows mean ± SEM Manders' coefficient of co-localization between $mGluR_5$ and Lamp1 ($P= 0.0039$; n = 3-4; paired Student's *t*-test). Scale bar 5 µm.

FIGURE S4

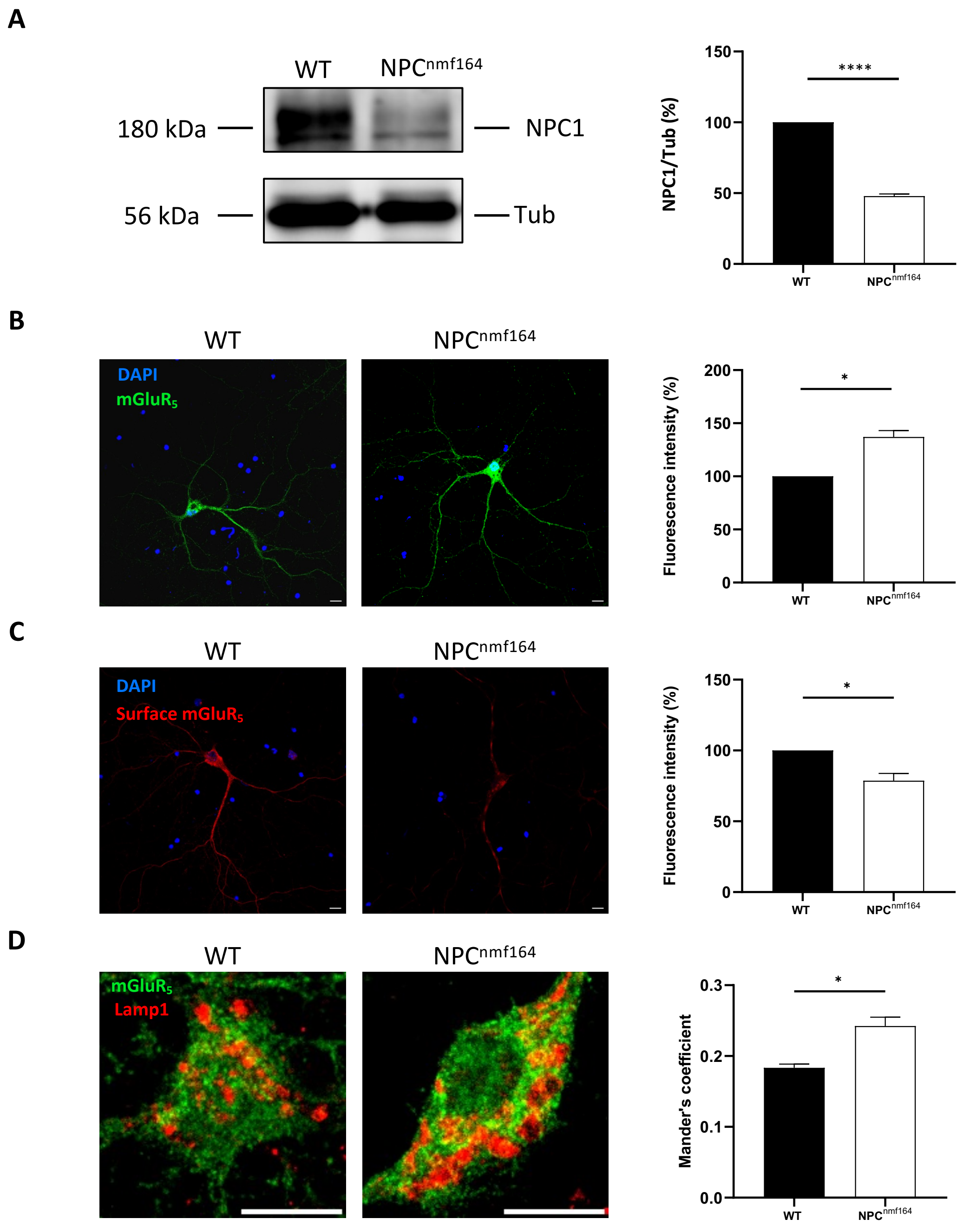

Figure S4. mGluR5 alterations in primary cultured neurons derived from Npc1^{nmf164} mice

A. Western blot showing NPC1 and tubulin (Tub) levels in cultured neurons from wt and Npc1^{nmf164} mice. Graph shows mean ± SEM levels of NPC1 normalized to Tub as a percentage of the values obtained in the wt neurons ($P < 0.0001$); n = 3; Unpaired two-tailed t-test).

B.C. IFA of total (B) and surface (C) mGluR$_5$ in cultured neurons from wt and Npc1^{nmf164} mice. Graphs show mean ± SEM fluorescence intensity associated with mGluR$_5$ as a percentage of the values obtained in the wt neurons ($P_B = 0.0432$; $P_C = 0.0483$ n = 3; Paired two-tailed t-tests). Scale bar 10 µm.

D. IFA of mGluR$_5$ and Lamp1 in cultured neurons from wt and Npc1^{nmf164} mice. Graph shows mean ± SEM Manders' coefficient of co-localization between mGluR$_5$ and Lamp1 (P= 0.0203; n = 3; Paired two-tailed t-tests). Scale bar 5 µm.

FIGURE S5

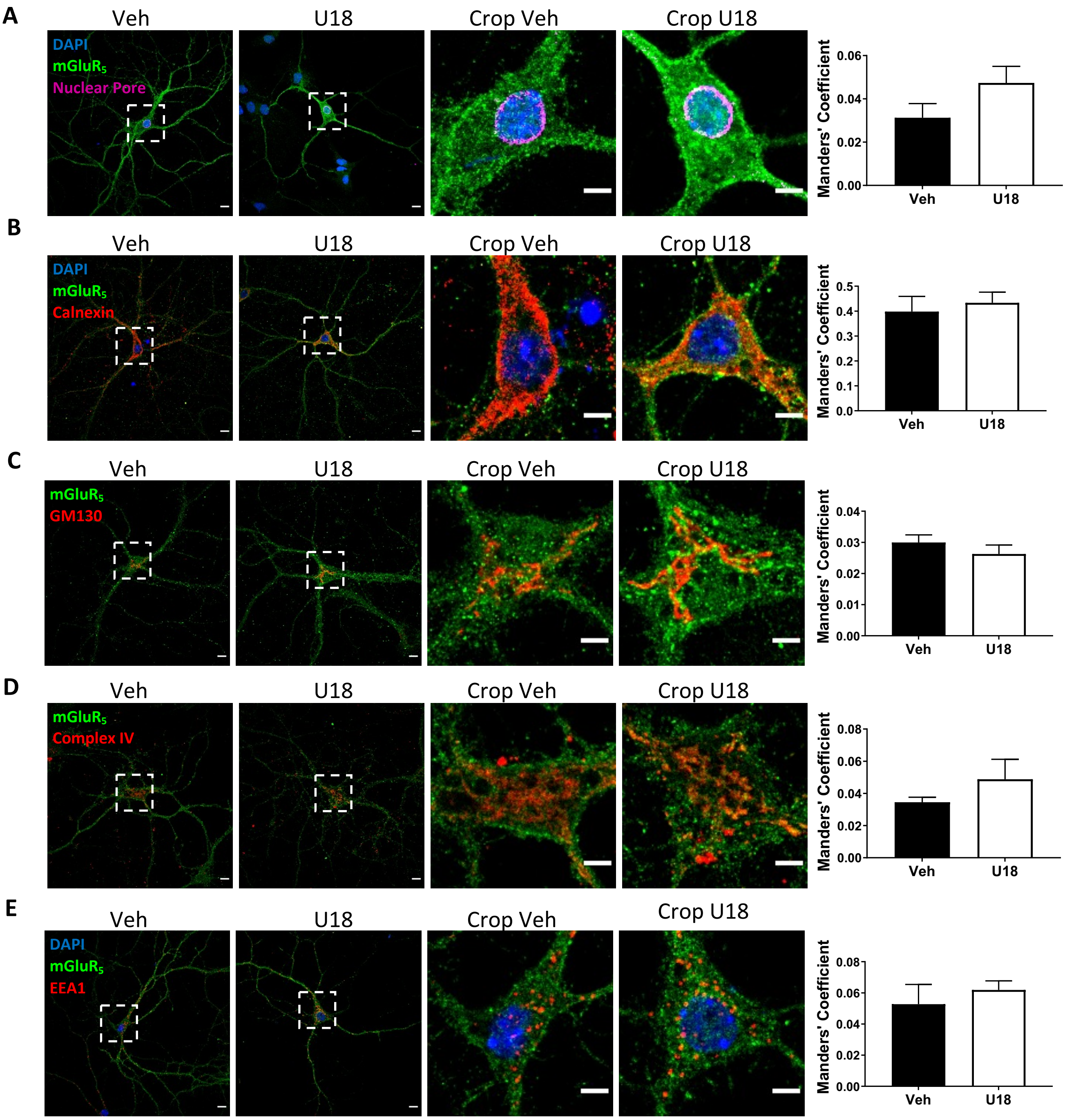

Figure S5. mGluR5 subcellular distribution in wt and NPC1-deficient cultured neurons

Immunofluorescences analysis of $mGluR_5$ co-stained with markers for nucleus (nuclear pore, **A**), endoplasmic reticulum (calnexin, **B**), Golgi apparatus (GM130, **C**), mitochondria (complex IV, **D**) and early endosomes (EEA1, **E**) in cultured neurons from wt mice treated with vehicle (Veh) or with U18 (NPC1 inhibitor). Graphs show mean ± SEM Manders' coefficient of co-localization between $mGluR_5$ and each of the markers for the indicated subcellular compartments (n = 5-6; paired Student's *t*-test). Scale bar 10 µm (5 µm in cropped images).

FIGURE S6

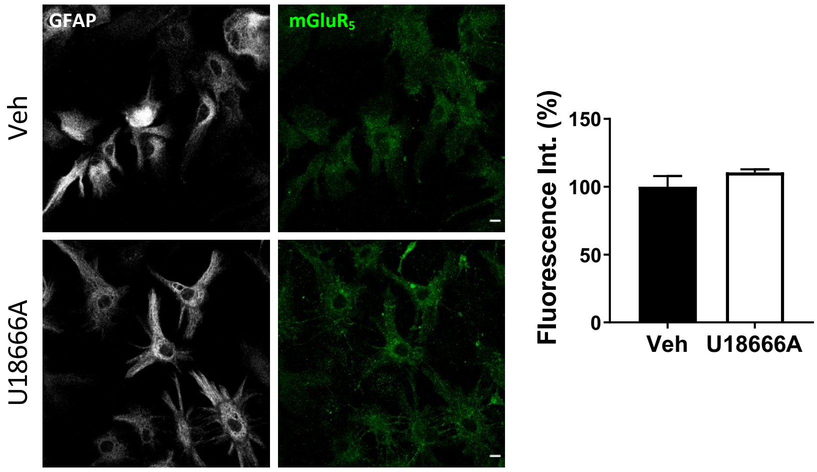

Figure S6. mGluR5 levels in wt and NPC1-deficient cultured astrocytes

Immunofluorescence analysis of $mGluR_5$ (green) and GFAP (white, marker of astrocytes) in cortical cultures from wt mice treated with vehicle (Veh) or with U18 (NPC1 inhibitor). Graph shows mean ± SEM fluorescence intensity associated with $mGluR_5$ as a percentage of the values obtained in the Veh-treated astrocytes (n = 3; paired Student's *t*-test). Scale bar 10 µm.

FIGURE S7

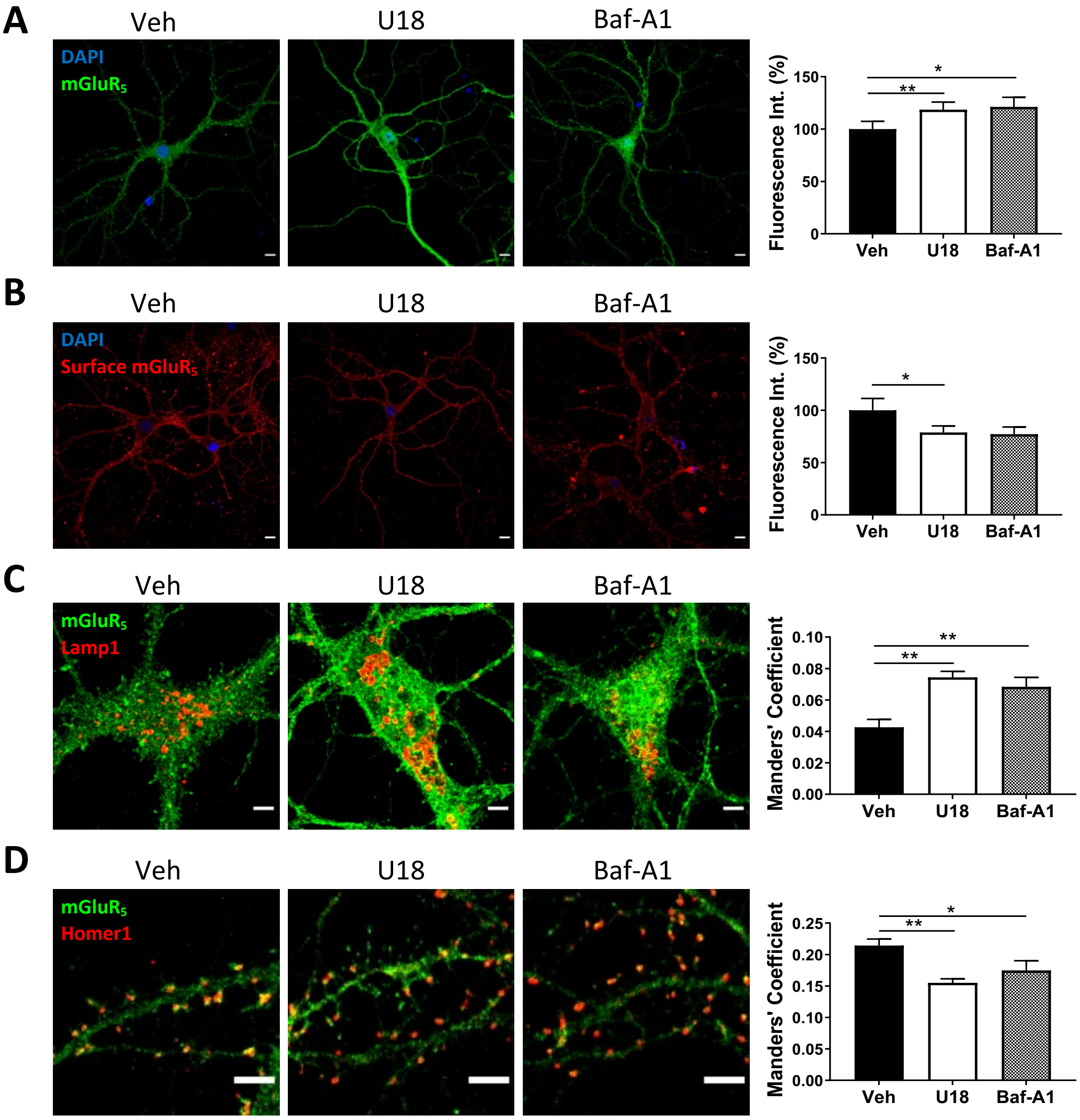

Figure S7. mGluR5 distribution after lysosomal inhibition

A,B. Immunofluorescence analysis (IFA) of total (a) and surface (b) $mGluR_5$ in cultured neurons from wt mice treated with vehicle (Veh), U18 (NPC1 inhibitor) or with bafilomycin A1 (Baf-A1; inhibitor of lysosomal function). Graphs shows mean ± SEM fluorescence intensity associated with $mGluR_5$ as a percentage of the values obtained in Veh-treated neurons ($P_{A.\ Veh\text{-}U18} = 0.0093$; $P_{A.\ Veh\text{-}Baf\text{-}A1} = 0.0435$; $P_{B.\ Veh\text{-}U18} = 0.0265$; n = 5; grouped one-way ANOVA Bonferroni *post hoc*). Scale bar 10 µm.

C,D. IFA showing co-staining of $mGluR_5$ with either Lamp1 (c) or Homer1 (d) in cultured neurons from wt mice treated with Veh, U18, or Baf-A1. Graphs show mean ± SEM Manders' coefficient of co-localization between $mGluR_5$ and Lamp1 or Homer1 ($P_{C.\ Veh\text{-}U18} = 0.0034$; $P_{C.\ Veh\text{-}Baf\text{-}A1} = 0.0017$; $P_{D.\ Veh\text{-}U18} = 0.0029$; $P_{D.\ Veh\text{-}Baf\text{-}A1} = 0.0291$; n = 5; grouped one-way ANOVA Bonferroni *post hoc*). Scale bar 5 µm.

FIGURE S8

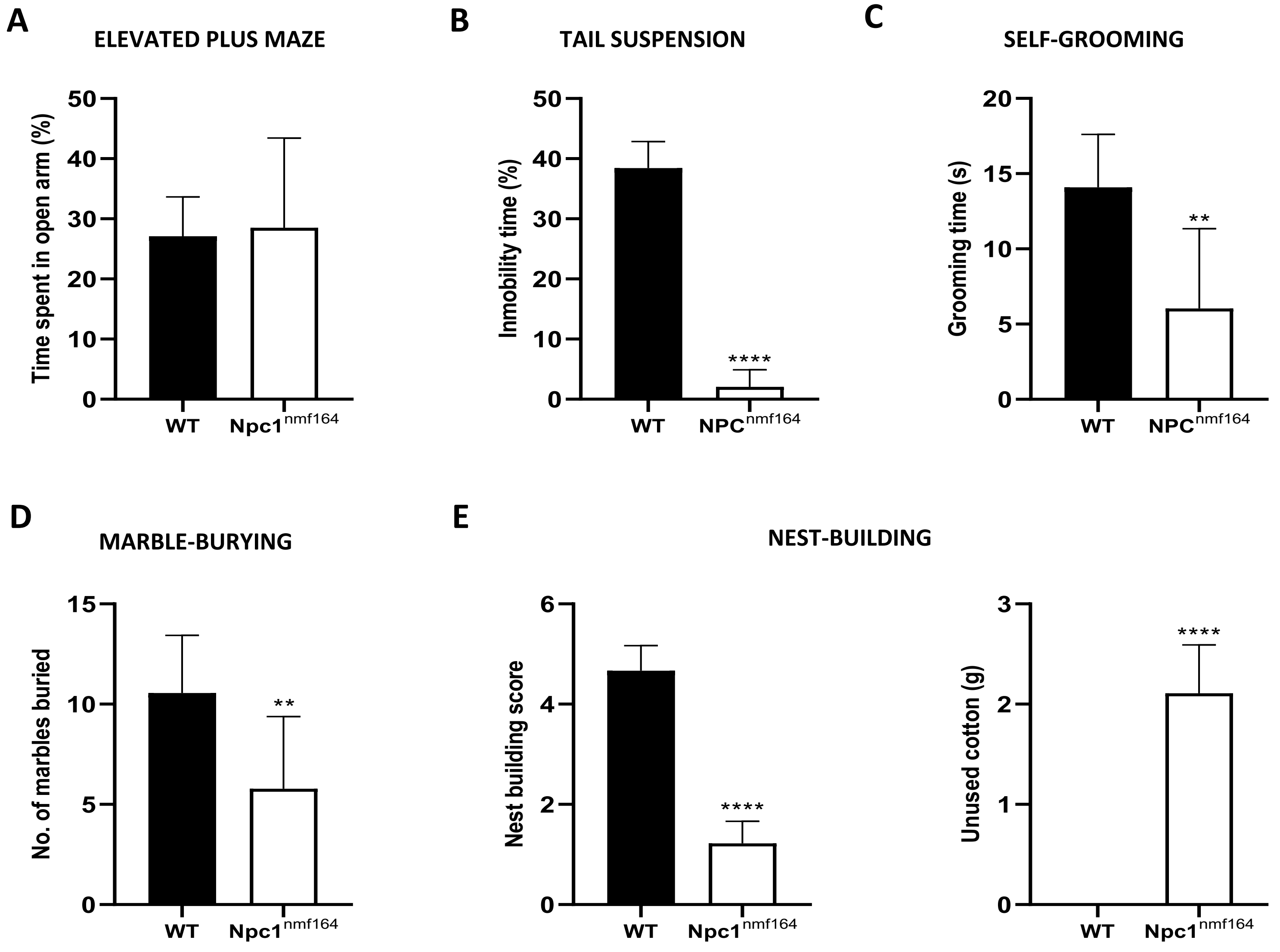

Figure S8. Behavioural alterations in Npc1^{nmf164} mice at 2.5 months of age

A. Graph showing mean ± SEM time spent in the open arm of the elevated plus maze as a percentage of the total exploring time by wt or Npc1^{nmf164} mice ($P < 0.8201$; n = 7-8; Student's *t*-test).

B. Graph showing mean ± SEM immobility time spent in the tail suspension test as a percentage of the total time by wt or Npc1^{nmf164} mice ($P < 0.0001$; n = 8-9; Student's *t*-test).

C. Graph showing mean ± SEM of time spent self-grooming by wt or Npc1^{nmf164} mice ($P < 0.0030$; n = 8; Student's *t*-test).

D. Graph showing mean ± SEM of number of marbles buried by wt or Npc1^{nmf164} mice ($P < 0.0067$; n = 9; Student's *t*-test).

E. Graphs show mean ± SEM of nest-building test score (left) and unused cotton material in grams (right) by wt or Npc1^{nmf164} mice in the nest-building test ($P_{left} < 0.0001$; $P_{right} < 0.0001$ n = 9; Student's *t*-test).

FIGURE S9

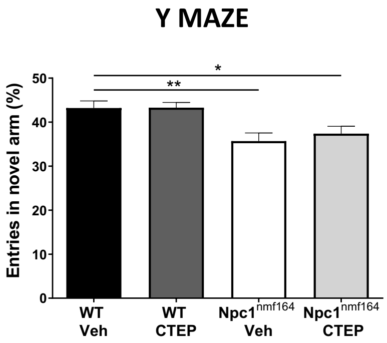

Figure S9. CTEP treatment does not ameliorate memory deficits in Npc1^{nmf164} mice

Graph showing mean ± SEM number of entries in the novel arm of the Y maze as a percentage of the total entries in all arms in wt or Npc1^{nmf164} mice treated with vehicle (Veh) or with CTEP (membrane-permeable mGluR$_5$ antagonist) (P = 0.0046; n = 8-13; grouped one-way ANOVA Bonferroni *post hoc*).
