# Supplementary material for: Enhanced mGluR5 intracellular activity causes psychiatric alterations in Niemann Pick type C disease
Source: Cell Death Dis. 2024 Oct 23;15(10):771. doi: 10.1038/s41419-024-07158-8 (PMC11499878; doi:10.1038/s41419-024-07158-8)

# FIGURE S10

**Figure S10A: Uncropped western blots from Figure 1A**

WB were cropped in two parts to quantify in the same membrane mGluR5 (upper part, red arrows indicate the band at 130KDa corresponding to mGluR5) in hippocampus, cortex and cerebellum preparations, and the loading control GAPDH (lower part). Numbers to the left indicate molecular weights in kDa.

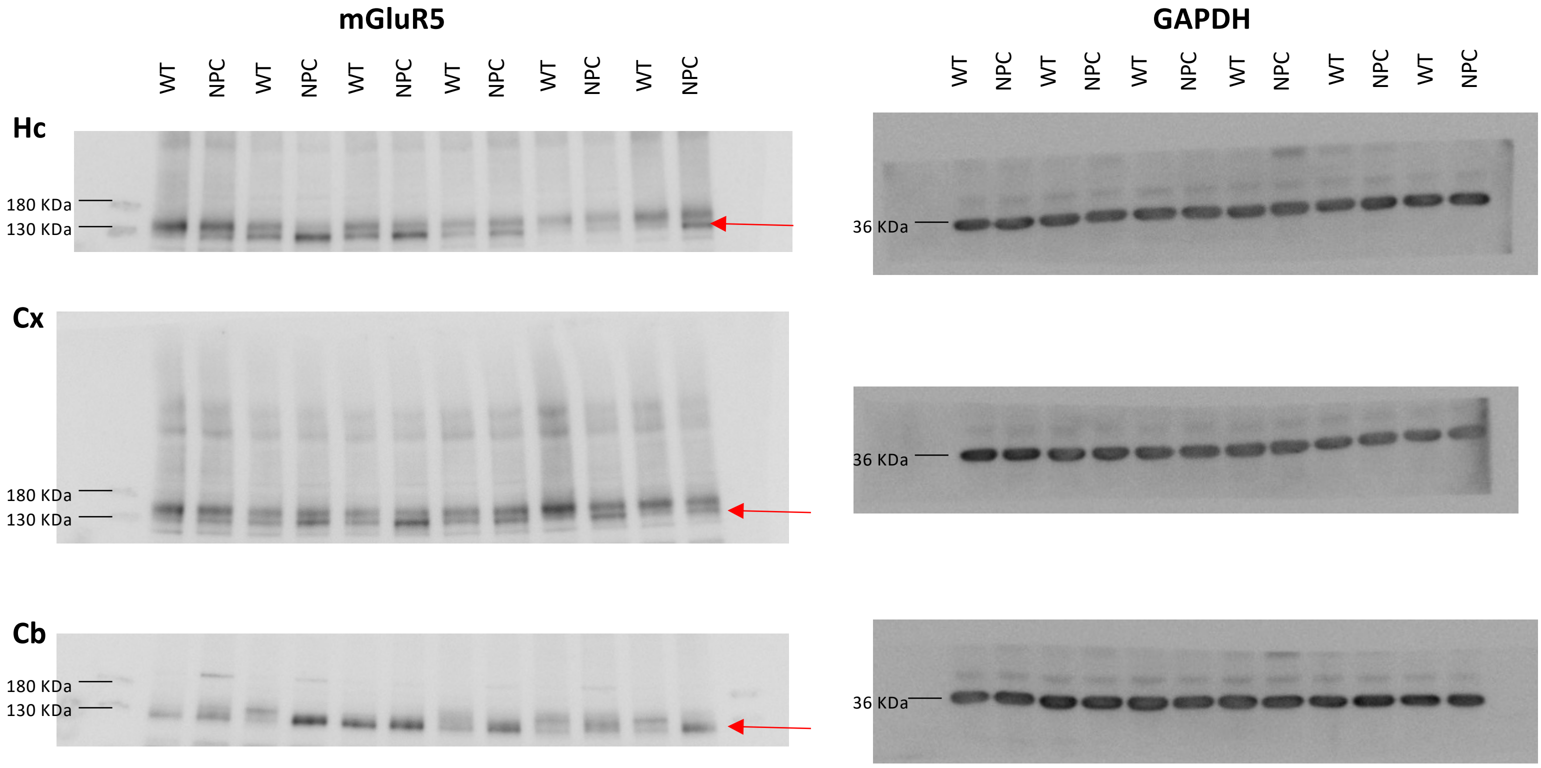


**Figure S10B: Uncropped WB from Figure 1G**

WB were cropped in two parts to quantify in the same membrane mGluR5 (upper part, red arrows indicate the band at 130KDa corresponding to mGluR5) in lysosome enriched whole brain preparations. The loading control used to normalize the values was Lamp1 (lower part). Numbers to the left indicate molecular weights in kDa. Lines marked with a red X were excluded from the quantification due to lack of genotype confirmation of the mice corresponding to said lines.

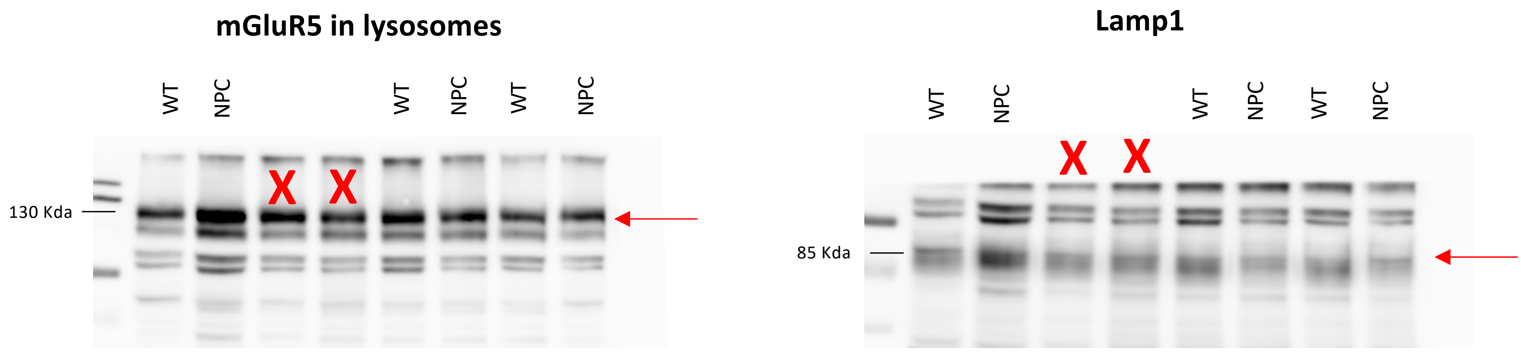

Supplement: Supplementary file 2 — Figure S10, Uncropped blots [file 41419_2024_7158_MOESM2_ESM.pdf]
